# 4-Chloro-*N*-(4-nitro­benzo­yl)benzene­sulfonamide

**DOI:** 10.1107/S160053681100969X

**Published:** 2011-03-19

**Authors:** P. A. Suchetan, Sabine Foro, B. Thimme Gowda

**Affiliations:** a Department of Chemistry, Mangalore University, Mangalagangotri 574 199, Mangalore, India; b Institute of Materials Science, Darmstadt University of Technology, Petersenstrasse 23, D-64287 Darmstadt, Germany

## Abstract

In the crystal structure of the title compound, C_13_H_9_ClN_2_O_5_S, the N—H bond is *trans* to the C=O bond (H—N—C—O torsion angle = 158.4°). The dihedral angle between the two aromatic rings is 87.8 (1)°. In the crystal, mol­ecules are linked into chains along the *b* axis *via* N—H⋯O hydrogen bonds.

## Related literature

For a study of the effect of substituents on the structures of *N*-(ar­yl)-amides, see: Gowda *et al.* (2000 ▶). For the effect of substituents in *N*-(ar­yl)-methane­sulfonamides, see: Gowda *et al.* (2007 ▶). For the effect of substituents on the structures of *N*-(*p*-substituted-benzo­yl)-*p*-substituted-benzene­sulfonamides, see: Suchetan *et al.* (2010 ▶, 2011 ▶).
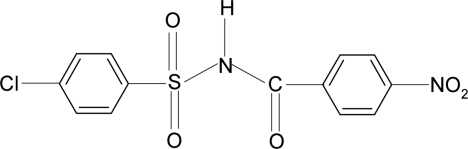


## Experimental

### 

#### Crystal data

C_13_H_9_ClN_2_O_5_S
*M* *_r_* = 340.73
Monoclinic, 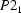

*a* = 11.713 (2) Å
*b* = 5.0681 (7) Å
*c* = 12.476 (2) Å
β = 104.45 (1)°
*V* = 717.2 (2) Å^3^
*Z* = 2
Mo *K*α radiation
μ = 0.44 mm^−1^
*T* = 293 K
0.32 × 0.18 × 0.06 mm

#### Data collection

Oxford Diffraction Xcalibur diffractometer with a Sapphire CCD detector
Absorption correction: multi-scan (*CrysAlis RED*; Oxford Diffraction, 2009 ▶) *T*_min_ = 0.873, *T*_max_ = 0.974
2688 measured reflections
2221 independent reflections
2052 reflections with *I* > 2σ(*I*)
*R*_int_ = 0.013

#### Refinement

*R*[*F* ^2^ > 2σ(*F* ^2^)] = 0.029
*wR*(*F* ^2^) = 0.071
*S* = 0.97
2221 reflections
202 parameters
2 restraints
H atoms treated by a mixture of independent and constrained refinement
Δρ_max_ = 0.19 e Å^−3^
Δρ_min_ = −0.22 e Å^−3^
Absolute structure: Flack (1983 ▶), 581 Friedel pairs
Flack parameter: 0.10 (8)

### 

Data collection: *CrysAlis CCD* (Oxford Diffraction, 2009 ▶); cell refinement: *CrysAlis RED* (Oxford Diffraction, 2009 ▶); data reduction: *CrysAlis RED*; program(s) used to solve structure: *SHELXS97* (Sheldrick, 2008 ▶); program(s) used to refine structure: *SHELXL97* (Sheldrick, 2008 ▶); molecular graphics: *PLATON* (Spek, 2009 ▶); software used to prepare material for publication: *SHELXL97*.

## Supplementary Material

Crystal structure: contains datablocks I, global. DOI: 10.1107/S160053681100969X/bt5491sup1.cif

Structure factors: contains datablocks I. DOI: 10.1107/S160053681100969X/bt5491Isup2.hkl

Additional supplementary materials:  crystallographic information; 3D view; checkCIF report

## Figures and Tables

**Table 1:** Hydrogen-bond geometry (Å, °)

*D*—H⋯*A*	*D*—H	H⋯*A*	*D*⋯*A*	*D*—H⋯*A*
N1—H1*N*⋯O2^i^	0.84 (2)	2.24 (2)	3.054 (3)	162 (3)

Symmetry code: (i) 
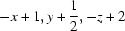
.

**Fig. 1.**
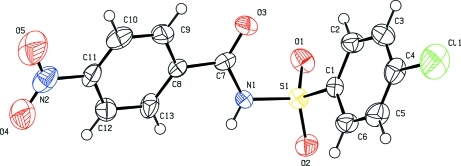
Molecular structure of the title compound, showing the atom- labelling scheme. Displacement ellipsoids are drawn at the 50% probability level.

**Fig. 2.**
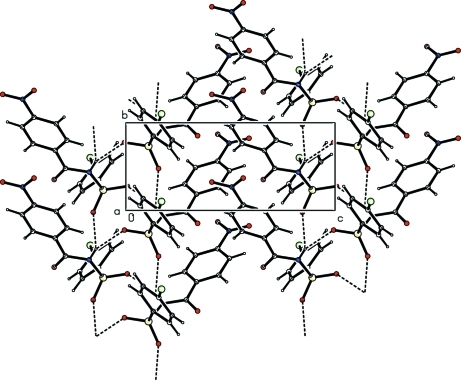
Molecular packing in the title compound. Hydrogen bonds are shown as dashed lines.

## supplementary crystallographic information

### Comment

The amide and sulfonamide moieties are important constituents of many biologically importantant compounds. As a part of studying the effect of substituents on the structures of this class of compounds (Gowda *et al.*, 2000, 2007; Suchetan *et al.*, 2010, 2011), the structure of 4-chloro-*N*-(4-nitrobenzoyl)-benzenesulfonamide has been determined (Fig.1). The conformation of the N—C bond in the C—SO_2_—NH—C(O) segment has *gauche* torsions with respect to the S═O bonds. Further, the conformation of the N—H bond in this segment is *anti* to the C=O bond, similar to those observed in *N*-(4-chlorobenzoyl)-4-chlorobenzenesulfonamide (II) (Suchetan *et al.*, 2010) and 4-methyl-*N*-(4-nitrobenzoyl)- benzenesulfonamide (III)(Suchetan *et al.*, 2011).

The molecules are twisted at the *S* atoms with the C—S(O_2_)—NH—C(O) torsional angle of 57.7 (2)°, compared to the values of 67.5 (3)° in (II) and 58.7 (3)° in (III).

The dihedral angle between the sulfonyl benzene ring and the —SO_2_—NH—C—O segment is 79.5 (1)°, compared to the values of 79.0 (1)° in (II) and 81.5 (2)° in (III).

The dihedral angle between the sulfonyl and the benzoyl benzene rings is 87.8 (1)°, compared to the values of 85.6 (1)° in (II) and 89.8 (1)° in (III).

The packing of molecules in the crystal linked by of pairs of N—H···O hydrogen bonds (Table 1) is shown in Fig. 2.

### Experimental

The title compound was prepared by refluxing a mixture of 4-nitrobenzoic acid, 4-chlorobenzenesulfonamide and phosphorous oxychloride for 3 hr on a water bath. The resultant mixture was cooled and poured into ice cold water. The solid obtained was filtered, washed thoroughly with water and then dissolved in sodium bicarbonate solution. The compound was later reprecipitated by acidifying the filtered solution with dilute HCl. It was filtered, dried and recrystallized.

Prism like colourless single crystals of the title compound used in X-ray diffraction studies were obtained by slow evaporation of its toluene solution at room temperature.

### Refinement

The H atom of the NH group was located in a difference map and later restrained to N—H = 0.86 (2) %A. The other H atoms were positioned with idealized geometry using a riding model with the aromatic C—H distance = 0.93 Å and methyl C—H = 0.96 Å. All H atoms were refined with isotropic displacement parameters set to 1.2 times of the *U*_eq_ of the parent atom.

### Crystal data

**Table:**

C_13_H_9_ClN_2_O_5_S	*F*(000) = 348
*M**_r_* = 340.73	*D*_x_ = 1.578 Mg m^−^^3^
Monoclinic, *P*2_1_	Mo *K*α radiation, λ = 0.71073 Å
Hall symbol: P 2yb	Cell parameters from 1764 reflections
*a* = 11.713 (2) Å	θ = 2.8–27.8°
*b* = 5.0681 (7) Å	µ = 0.44 mm^−^^1^
*c* = 12.476 (2) Å	*T* = 293 K
β = 104.45 (1)°	Prism, colourless
*V* = 717.2 (2) Å^3^	0.32 × 0.18 × 0.06 mm
*Z* = 2	

### Data collection

**Table:**

Oxford Diffraction Xcalibur diffractometer with a Sapphire CCD detector	2221 independent reflections
Radiation source: fine-focus sealed tube	2052 reflections with *I* > 2σ(*I*)
graphite	*R*_int_ = 0.013
Rotation method data acquisition using ω and φ scans	θ_max_ = 26.4°, θ_min_ = 2.8°
Absorption correction: multi-scan (*CrysAlis RED*; Oxford Diffraction, 2009)	*h* = −11→14
*T*_min_ = 0.873, *T*_max_ = 0.974	*k* = −6→5
2688 measured reflections	*l* = −15→13

### Refinement

**Table:**

Refinement on *F*^2^	Secondary atom site location: difference Fourier map
Least-squares matrix: full	Hydrogen site location: inferred from neighbouring sites
*R*[*F*^2^ > 2σ(*F*^2^)] = 0.029	H atoms treated by a mixture of independent and constrained refinement
*wR*(*F*^2^) = 0.071	*w* = 1/[σ^2^(*F*_o_^2^) + (0.0265*P*)^2^ + 0.5326*P*] where *P* = (*F*_o_^2^ + 2*F*_c_^2^)/3
*S* = 0.97	(Δ/σ)_max_ = 0.031
2221 reflections	Δρ_max_ = 0.19 e Å^−^^3^
202 parameters	Δρ_min_ = −0.22 e Å^−^^3^
2 restraints	Absolute structure: Flack (1983), 581 Friedel pairs
Primary atom site location: structure-invariant direct methods	Flack parameter: 0.10 (8)

### Special details

**Table:**

Experimental. CrysAlis RED (Oxford Diffraction, 2009) Empirical absorption correction using spherical harmonics, implemented in SCALE3 ABSPACK scaling algorithm.
Geometry. All e.s.d.'s (except the e.s.d. in the dihedral angle between two l.s. planes) are estimated using the full covariance matrix. The cell e.s.d.'s are taken into account individually in the estimation of e.s.d.'s in distances, angles and torsion angles; correlations between e.s.d.'s in cell parameters are only used when they are defined by crystal symmetry. An approximate (isotropic) treatment of cell e.s.d.'s is used for estimating e.s.d.'s involving l.s. planes.
Refinement. Refinement of *F*^2^ against ALL reflections. The weighted *R*-factor *wR* and goodness of fit *S* are based on *F*^2^, conventional *R*-factors *R* are based on *F*, with *F* set to zero for negative *F*^2^. The threshold expression of *F*^2^ > σ(*F*^2^) is used only for calculating *R*-factors(gt) *etc*. and is not relevant to the choice of reflections for refinement. *R*-factors based on *F*^2^ are statistically about twice as large as those based on *F*, and *R*- factors based on ALL data will be even larger.

### Fractional atomic coordinates and isotropic or equivalent isotropic displacement parameters (Å^2^)

**Table:**

	*x*	*y*	*z*	*U*_iso_*/*U*_eq_	
C1	0.7268 (2)	0.3471 (6)	0.8688 (2)	0.0313 (6)	
C2	0.7745 (2)	0.2212 (8)	0.7918 (2)	0.0410 (6)	
H2	0.7339	0.0840	0.7493	0.049*	
C3	0.8836 (3)	0.3014 (7)	0.7785 (2)	0.0489 (9)	
H3	0.9174	0.2178	0.7277	0.059*	
C4	0.9402 (3)	0.5060 (7)	0.8417 (3)	0.0473 (8)	
C5	0.8926 (3)	0.6356 (7)	0.9171 (3)	0.0487 (8)	
H5	0.9324	0.7760	0.9579	0.058*	
C6	0.7843 (3)	0.5541 (6)	0.9314 (2)	0.0400 (7)	
H6	0.7510	0.6378	0.9826	0.048*	
C7	0.4676 (2)	0.4805 (6)	0.7118 (2)	0.0325 (6)	
C8	0.3785 (2)	0.6855 (5)	0.66280 (19)	0.0305 (7)	
C9	0.3743 (3)	0.7628 (8)	0.5547 (2)	0.0465 (7)	
H9	0.4248	0.6857	0.5168	0.056*	
C10	0.2952 (3)	0.9537 (7)	0.5037 (2)	0.0511 (9)	
H10	0.2930	1.0083	0.4320	0.061*	
C11	0.2195 (3)	1.0625 (6)	0.5603 (2)	0.0419 (7)	
C12	0.2217 (3)	0.9902 (7)	0.6672 (2)	0.0464 (8)	
H12	0.1702	1.0665	0.7041	0.056*	
C13	0.3026 (2)	0.8007 (6)	0.7184 (2)	0.0430 (8)	
H13	0.3058	0.7504	0.7908	0.052*	
N1	0.48802 (19)	0.4379 (5)	0.82564 (17)	0.0292 (5)	
H1N	0.471 (2)	0.560 (5)	0.865 (2)	0.035*	
N2	0.1351 (2)	1.2683 (6)	0.5060 (2)	0.0551 (7)	
O1	0.56993 (19)	−0.0216 (4)	0.84448 (18)	0.0447 (5)	
O2	0.59606 (16)	0.2753 (5)	1.00622 (14)	0.0427 (5)	
O3	0.51982 (18)	0.3525 (5)	0.65776 (16)	0.0485 (6)	
O4	0.0734 (3)	1.3697 (6)	0.5600 (2)	0.0791 (9)	
O5	0.1323 (3)	1.3233 (6)	0.4105 (2)	0.0880 (10)	
Cl1	1.07887 (8)	0.6005 (3)	0.82955 (9)	0.0835 (4)	
S1	0.59243 (6)	0.23417 (14)	0.89175 (5)	0.03068 (15)	

### Atomic displacement parameters (Å^2^)

**Table:**

	*U*^11^	*U*^22^	*U*^33^	*U*^12^	*U*^13^	*U*^23^
C1	0.0300 (13)	0.0312 (15)	0.0316 (13)	−0.0001 (12)	0.0058 (10)	0.0047 (12)
C2	0.0413 (14)	0.0426 (17)	0.0402 (13)	−0.0036 (16)	0.0122 (11)	−0.0049 (17)
C3	0.0430 (16)	0.064 (3)	0.0437 (15)	0.0003 (16)	0.0182 (13)	−0.0025 (16)
C4	0.0324 (15)	0.061 (2)	0.0476 (17)	−0.0074 (15)	0.0083 (13)	0.0122 (17)
C5	0.0422 (16)	0.046 (2)	0.0529 (18)	−0.0138 (15)	0.0016 (14)	−0.0060 (16)
C6	0.0421 (15)	0.0353 (18)	0.0411 (15)	−0.0010 (13)	0.0077 (12)	−0.0040 (13)
C7	0.0315 (13)	0.0380 (16)	0.0280 (13)	−0.0067 (12)	0.0075 (10)	−0.0051 (13)
C8	0.0316 (12)	0.0320 (19)	0.0264 (11)	−0.0074 (11)	0.0045 (10)	0.0008 (11)
C9	0.0531 (17)	0.057 (2)	0.0314 (13)	0.0059 (18)	0.0139 (12)	0.0024 (16)
C10	0.066 (2)	0.055 (2)	0.0294 (14)	0.0004 (17)	0.0054 (14)	0.0114 (15)
C11	0.0419 (15)	0.0377 (18)	0.0382 (15)	−0.0029 (13)	−0.0047 (12)	0.0029 (13)
C12	0.0415 (16)	0.056 (2)	0.0424 (16)	0.0087 (15)	0.0125 (13)	0.0072 (16)
C13	0.0372 (14)	0.057 (2)	0.0352 (14)	0.0053 (14)	0.0095 (11)	0.0109 (14)
N1	0.0332 (12)	0.0273 (13)	0.0270 (11)	0.0010 (10)	0.0077 (9)	−0.0023 (9)
N2	0.0610 (17)	0.0388 (18)	0.0528 (15)	0.0008 (15)	−0.0094 (13)	0.0046 (16)
O1	0.0487 (12)	0.0259 (11)	0.0616 (13)	−0.0035 (9)	0.0176 (10)	−0.0016 (10)
O2	0.0467 (11)	0.0505 (15)	0.0321 (9)	0.0011 (10)	0.0119 (8)	0.0092 (10)
O3	0.0523 (12)	0.0615 (15)	0.0341 (10)	0.0126 (11)	0.0151 (9)	−0.0034 (10)
O4	0.083 (2)	0.0648 (19)	0.0776 (18)	0.0285 (16)	−0.0022 (15)	0.0016 (15)
O5	0.109 (2)	0.079 (2)	0.0632 (17)	0.0196 (17)	−0.0012 (15)	0.0318 (16)
Cl1	0.0452 (5)	0.1158 (9)	0.0939 (8)	−0.0266 (6)	0.0258 (5)	0.0049 (7)
S1	0.0341 (3)	0.0258 (3)	0.0332 (3)	−0.0019 (3)	0.0103 (2)	0.0029 (3)

### Geometric parameters (Å, °)

**Table:**

C1—C6	1.379 (4)	C8—C13	1.386 (4)
C1—C2	1.382 (4)	C9—C10	1.380 (5)
C1—S1	1.764 (3)	C9—H9	0.9300
C2—C3	1.389 (4)	C10—C11	1.379 (5)
C2—H2	0.9300	C10—H10	0.9300
C3—C4	1.369 (5)	C11—C12	1.377 (4)
C3—H3	0.9300	C11—N2	1.480 (4)
C4—C5	1.375 (5)	C12—C13	1.388 (4)
C4—Cl1	1.736 (3)	C12—H12	0.9300
C5—C6	1.387 (4)	C13—H13	0.9300
C5—H5	0.9300	N1—S1	1.655 (2)
C6—H6	0.9300	N1—H1N	0.843 (17)
C7—O3	1.206 (3)	N2—O5	1.216 (4)
C7—N1	1.397 (3)	N2—O4	1.218 (4)
C7—C8	1.491 (4)	O1—S1	1.421 (2)
C8—C9	1.394 (3)	O2—S1	1.4333 (18)
			
C6—C1—C2	121.3 (3)	C10—C9—H9	120.0
C6—C1—S1	119.0 (2)	C11—C10—C9	119.2 (3)
C2—C1—S1	119.6 (2)	C11—C10—H10	120.4
C3—C2—C1	119.4 (3)	C9—C10—H10	120.4
C3—C2—H2	120.3	C12—C11—C10	122.0 (3)
C1—C2—H2	120.3	C12—C11—N2	118.7 (3)
C4—C3—C2	118.7 (3)	C10—C11—N2	119.3 (3)
C4—C3—H3	120.6	C11—C12—C13	118.4 (3)
C2—C3—H3	120.6	C11—C12—H12	120.8
C5—C4—C3	122.3 (3)	C13—C12—H12	120.8
C5—C4—Cl1	118.4 (3)	C8—C13—C12	120.7 (3)
C3—C4—Cl1	119.2 (3)	C8—C13—H13	119.7
C4—C5—C6	119.1 (3)	C12—C13—H13	119.7
C4—C5—H5	120.5	C7—N1—S1	121.38 (18)
C6—C5—H5	120.5	C7—N1—H1N	118 (2)
C5—C6—C1	119.1 (3)	S1—N1—H1N	115 (2)
C5—C6—H6	120.4	O5—N2—O4	124.8 (3)
C1—C6—H6	120.4	O5—N2—C11	117.5 (3)
O3—C7—N1	120.2 (3)	O4—N2—C11	117.7 (3)
O3—C7—C8	123.1 (2)	O1—S1—O2	120.16 (14)
N1—C7—C8	116.7 (2)	O1—S1—N1	108.96 (13)
C9—C8—C13	119.6 (3)	O2—S1—N1	103.91 (12)
C9—C8—C7	116.4 (3)	O1—S1—C1	108.01 (14)
C13—C8—C7	124.0 (2)	O2—S1—C1	107.98 (12)
C8—C9—C10	120.0 (3)	N1—S1—C1	107.14 (12)
C8—C9—H9	120.0		
			
C6—C1—C2—C3	1.1 (5)	N2—C11—C12—C13	−178.8 (3)
S1—C1—C2—C3	−176.4 (2)	C9—C8—C13—C12	0.6 (4)
C1—C2—C3—C4	−0.7 (5)	C7—C8—C13—C12	−179.9 (3)
C2—C3—C4—C5	−0.4 (5)	C11—C12—C13—C8	−0.6 (5)
C2—C3—C4—Cl1	177.7 (3)	O3—C7—N1—S1	6.0 (4)
C3—C4—C5—C6	1.2 (5)	C8—C7—N1—S1	−174.85 (18)
Cl1—C4—C5—C6	−177.0 (2)	C12—C11—N2—O5	−177.8 (3)
C4—C5—C6—C1	−0.7 (5)	C10—C11—N2—O5	3.7 (5)
C2—C1—C6—C5	−0.4 (4)	C12—C11—N2—O4	2.0 (5)
S1—C1—C6—C5	177.1 (2)	C10—C11—N2—O4	−176.5 (3)
O3—C7—C8—C9	−12.6 (4)	C7—N1—S1—O1	−58.9 (2)
N1—C7—C8—C9	168.2 (3)	C7—N1—S1—O2	171.9 (2)
O3—C7—C8—C13	167.9 (3)	C7—N1—S1—C1	57.7 (2)
N1—C7—C8—C13	−11.3 (4)	C6—C1—S1—O1	−162.3 (2)
C13—C8—C9—C10	0.3 (5)	C2—C1—S1—O1	15.2 (3)
C7—C8—C9—C10	−179.3 (3)	C6—C1—S1—O2	−31.0 (3)
C8—C9—C10—C11	−1.1 (5)	C2—C1—S1—O2	146.6 (2)
C9—C10—C11—C12	1.2 (5)	C6—C1—S1—N1	80.4 (2)
C9—C10—C11—N2	179.6 (3)	C2—C1—S1—N1	−102.0 (2)
C10—C11—C12—C13	−0.4 (5)		

### Hydrogen-bond geometry (Å, °)

**Table:**

*D*—H···*A*	*D*—H	H···*A*	*D*···*A*	*D*—H···*A*
N1—H1N···O2^i^	0.84 (2)	2.24 (2)	3.054 (3)	162 (3)

Symmetry codes: (i) −*x*+1, *y*+1/2, −*z*+2.

## References

[bb1] Flack, H. D. (1983). *Acta Cryst.* A**39**, 876–881.

[bb2] Gowda, B. T., Foro, S. & Fuess, H. (2007). *Acta Cryst.* E**63**, o2597.

[bb3] Gowda, B. T., Paulus, H. & Fuess, H. (2000). *Z. Naturforsch. Teil A*, **55**, 791–800.

[bb4] Oxford Diffraction (2009). *CrysAlis CCD* and *CrysAlis RED* Oxford Diffraction Ltd, Yarnton, England.

[bb5] Sheldrick, G. M. (2008). *Acta Cryst.* A**64**, 112–122. 10.1107/S0108767307043930 18156677

[bb6] Spek, A. L. (2009). *Acta Cryst.* D**65**, 148–155. 10.1107/S090744490804362X PMC2631630 19171970

[bb7] Suchetan, P. A., Foro, S. & Gowda, B. T. (2011). *Acta Cryst.* E**67**, o917. 10.1107/S1600536811009470 PMC3100041 21754188

[bb8] Suchetan, P. A., Gowda, B. T., Foro, S. & Fuess, H. (2010). *Acta Cryst.* E**66**, o1253. 10.1107/S160053681001559X PMC2979647 21579357

